# Development of mesenchymal subtype gene signature for clinical application in gastric cancer

**DOI:** 10.18632/oncotarget.19985

**Published:** 2017-08-07

**Authors:** Jeeyun Lee, Razvan Cristescu, Kyoung-Mee Kim, Kyung Kim, Seung Tae Kim, Se Hoon Park, Won Ki Kang

**Affiliations:** ^1^ Division of Hematology-Oncology, Department of Medicine, Samsung Medical Center, Sungkyunkwan University School of Medicine, Seoul, Korea; ^2^ Department of Genetics and Pharmacogenomics (GpGx), Merck Research Laboratories, Merck Sharpe & Dohme, Boston, MA, USA; ^3^ Department of Pathology and Translational Genomics, Samsung Medical Center, Sungkyunkwan University School of Medicine, Seoul, Korea

**Keywords:** stomach neoplasm, genomics, gene signature, subtypes, mesenchymal

## Abstract

Previously, in the Asian Cancer Research Group (ACRG) project, we defined four distinct molecular subtypes in gastric cancer (GC). Mesenchymal (microsatellite stable with epithelial-to-mesenchymal transition phenotype, MSS/EMT) tumors showed the worst prognosis among all the subtypes. To develop a gene signature for predicting mesenchymal subtype GC, we conducted gene expression profiling using a NanoString assay in 70 ACRG specimens. The gene signature was validated in an independent set obtained from the prospective Adjuvant chemoRadioTherapy In Stomach Tumor (ARTIST) trial. The association between the mesenchymal subtype and survival was investigated. After cross-platform concordance test performed in 70 ACRG specimens, a 71-gene MSS/EMT signature was obtained. In the validation set, the gene signature predicted that 20 of 73 (27%) patients had mesenchymal tumors. Patients with mesenchymal subtype had diffuse GC, poorly-differentiated or signet ring cell carcinoma, and were microsatellite stable. The estimated hazard ratio for survival in patients with mesenchymal GC compared to those with non-mesenchymal tumors was 2.262 (95% confidence interval, 1.410 to 3.636; P=0.001). The survival difference remained significant when the subtypes were analyzed according to clinical prognostic parameters. This study suggested that the NanoString-based 71-gene signature for mesenchymal subtype is a strong predictor of the outcome in patients with GC.

## INTRODUCTION

Gastric cancer (GC) is one of the most frequently occurring malignancies worldwide and the third-leading cause of cancer death [1]. Most GC patients present with advanced stage disease and the overall prognosis remains very poor. Clinical trials involving novel targeted agents have demonstrated little success as palliative treatment for GC, with the exceptions of trastuzumab in patients with human epidermal growth factor receptor 2 (HER2)-positive tumors [2], and ramucirumab as a second-line treatment [3, 4]. Possible explanations for the lack of improvement in survival include that GC is a heterogeneous disease, with substantial differences in its aggressiveness and responsiveness to therapy, and its clinical outcome and prognosis in the individual patient do not always conform to the published data [5]. Subtypes with different prognosis and different effects on cancer therapy, if found, may help ensure that patients receive the best possible treatment, thereby avoiding unnecessary treatment and associated toxicities, to eventually improve the overall outcomes.

Beyond well-known morphological subtypes for GC [6], most recently, distinct molecularly defined subtypes have emerged in GC [6-10]. The Asian Cancer Research Group (ACRG) was founded as a non-profit consortium of the pharmaceutical industry, academic medical centers, and sequencing companies to characterize GC subtypes. Molecular classification by the ACRG demonstrated that there are four subtypes: 1) GC with microsatellite instability (MSI); 2) GC with microsatellite stable (MSS) with an epithelial-to-mesenchymal transition (EMT) phenotype; 3) GC with a p53 signature (expressing *CDKN1A* and *MDM2*); or 4) tumors without the p53 signature. The most striking finding of this analysis was that the MSS/EMT subtype showed a significantly higher recurrence rate, higher probability of developing peritoneal seeding at the first site of recurrence, younger age at diagnosis, and extremely poor survival compared to other subtypes [8]. The survival curve consistently declines over 5 years because of disease recurrence leading to death. Hence, more aggressive treatment should be developed for this subset of GC to improve survival.

In order to make a gene expression profiling-based molecular classification more clinically applicable, we developed a gene signature system involving NanoString-based targeted expression profiling to: 1) investigate the concordance rate between gene expression levels using conventional versus targeted gene expression profiling using the NanoString assay for the mesenchymal MSS/EMT subtype in 70 randomly selected samples from the ACRG; 2) define cross-platform concordance with the nCounter assay for MSS/EMT signature; 3) test the mesenchymal NanoString assay in 70 ACRG samples with known molecular subtypes; 4) validate the mesenchymal gene signature in the 73 samples obtained from the prospective phase III Adjuvant chemoRadioTherapy In Stomach Tumor (ARTIST) trial [11, 12].

## RESULTS

### Development of mesenchymal subtype signature

**Figure 1:**
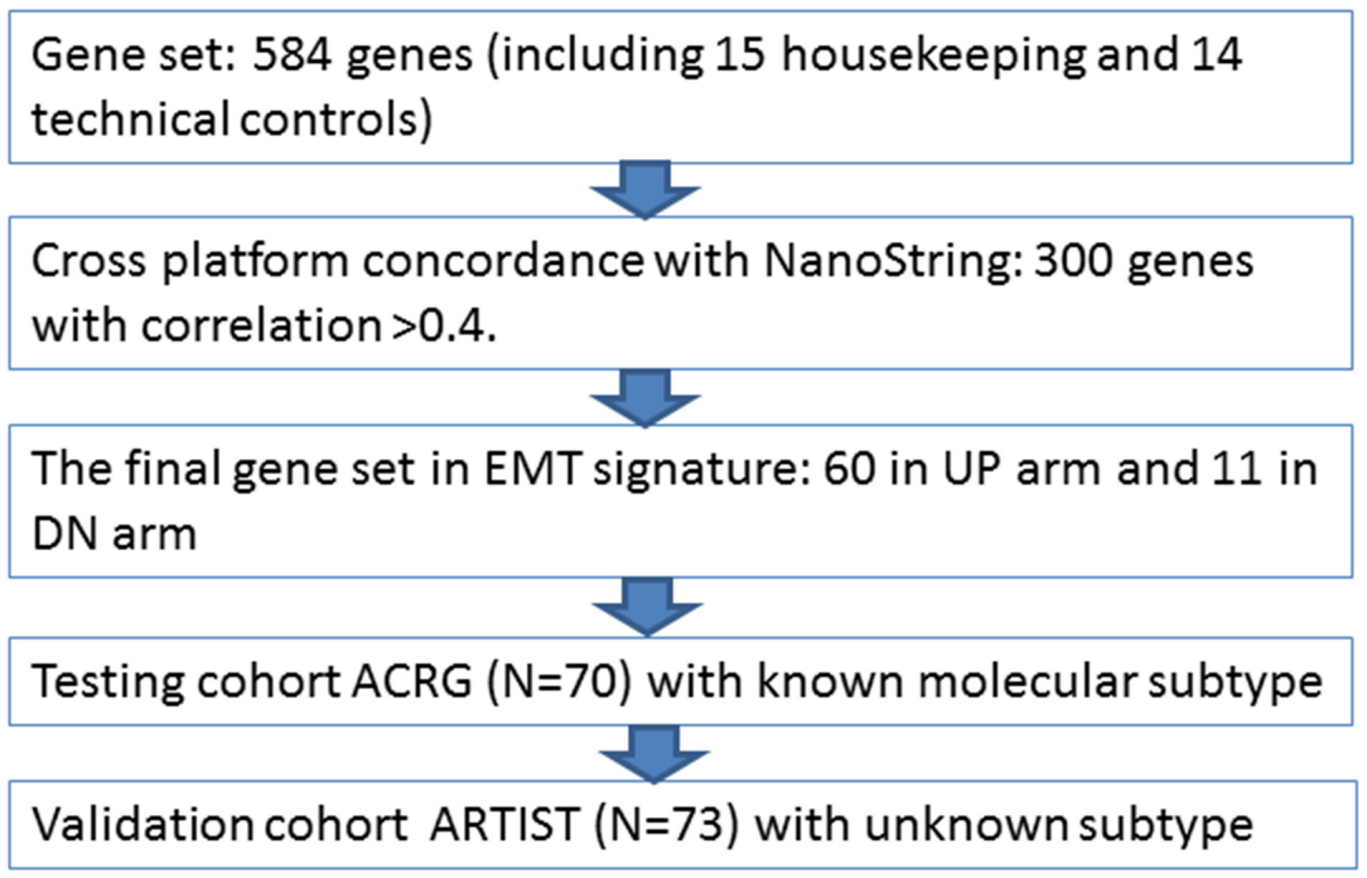
Study design to explore and validate gene signature for mesenchymal subtype EMT, epithelial-to-mesenchymal transition; ACRG, Asian Cancer Research Group; ARTIST, Adjuvant chemoRadiotherapy In Stomach Tumor.

**Figure 2:**
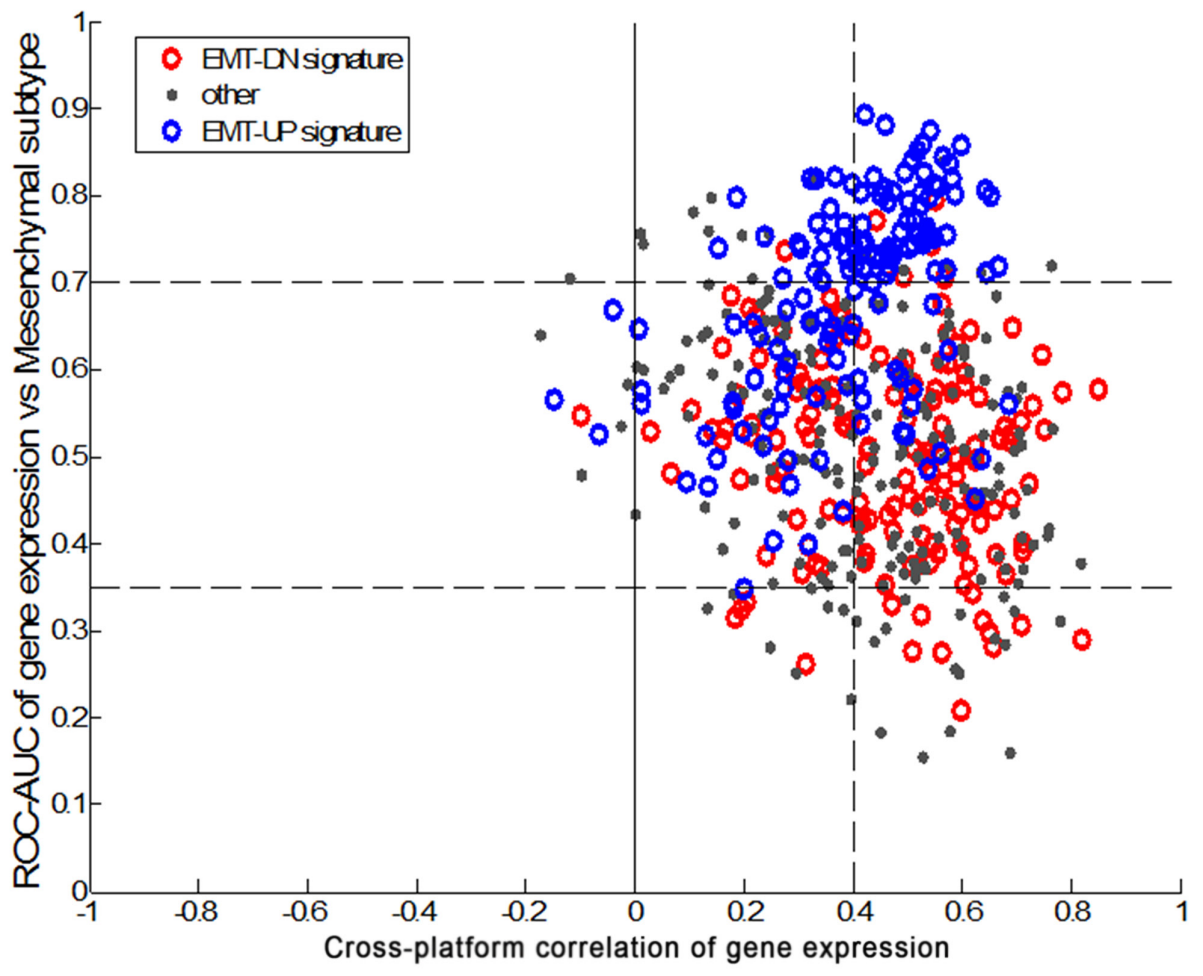
Concordance test subtypes classified by Affymetrix gene expression profiling and mesenchymal subtype by NanoString

A total of 143 tumor specimens were analyzed: 70 and 73 patients from the ACRG and the ARTIST cohort, respectively. As expected, the ARTIST patients were younger and had earlier stage disease than those in the ACRG cohort (Table 1). The study design is outlined in Figure 1. In brief, we began the cross-platform concordance test using 70 ACRG tissue specimens with NanoString targeted gene expression. After refining the final gene set, the concordance was tested between subtypes classified by Affymetrix and mesenchymal subtype by NanoString. As shown in Figure 2, 60 genes were upregulated from the EMT/MSS gene signature, whereas 11 genes were downregulated, revealing a high correlation between the two platforms. Finally, the mesenchymal subtype in the ARTIST cohort was evaluated to determine whether the gene set could predict the clinical features of MSS/EMT. We chose quartile-based cutoffs (top quartile) for each dataset (0.325 for the ARTIST and 0.14 for the ACRG).

**Table 1:** Clinical characteristics of study participants

	ACRG (N=70)	ARTIST (N=73)
Age, years		
Median	63	51
Range	25 to 78	35 to 76
Gender		
Male	51	46
Female	19	27
Tumor stage		
1-2	16	36
3-4	54	37
Lauren classification		
Intestinal	27	15
Diffuse	36	53
Mixed or not available	7	5
MSI high	19 (27%)	7 (15%)
Mesenchymal subtype	13 (19%)	20 (27%)

ACRG, Asian Cancer Research Group; ARTIST, Adjuvant chemoRadiotherapy In Stomach Tumor; MSI, microsatellite instability.

Next, we tested the 71-gene EMT/MSS signature in the ACRG cohort with known molecular subtypes using the conventional Affymetrix method. The concordance rate between the two platforms were very high: among 70 ACRG samples, only two samples which were previously categorized as mesenchymal subtype by Affymetrix platform were classified as non-mesenchymal subtype by NanoString (Table 2). There were 16 MSS/EMT, 20 MSI, 23 P53 active/MSS, and 11 P53 inactive/MSS subtypes included in the cohort. Of the 16 MSS/EMT samples, 14 (88%) were identified as mesenchymal subtype by NanoString. Of note, these two NanoString non-mesenchymal but MSS/EMT tumors were of signet ring cell subtype (ACRG #42, #47). Histologic review revealed that the #42 subjected to ACRG analysis was obtained from serosal side, whereas the NanoString specimen contained tumors from gastric mucosa. Similarly, ACRG #47 tumor contained a mixture of signet ring cell carcinoma and tubular moderately-differentiated adenocarcinoma. All samples from MSI, P53 active/MSS, P53 inactive/MSS ACRG subtypes were categorized as non-mesenchymal with 100% concordance based on our scoring system.

**Table 2:** Concordance between ACRG subtype classification by Affymetrix gene expression and targeted gene expression by NanoString

Sample #	Mesenchymal	ACRG subtype		EBV-ISH	MSI	MLH1 by IHC	Lauren classification	Pathology	Stage
**ACRG#1**	non-mesenchymal	MSI	-0.17	negative	MSS	partial loss	intestinal	moderately differentiated adenocarcinoma	IB
**ACRG#2**	mesenchymal	MSS/EMT	0.32	negative	MSS	preserved	diffuse	signet ring cell carcinoma	III
**ACRG#3**	mesenchymal	MSS/EMT	0.37	positive	MSS	preserved	diffuse	poorly differentiated adenocarcinoma	III
**ACRG#4**	mesenchymal	MSS/EMT	0.53	negative	MSS	preserved	mixed	mucinous adenocarcinoma	II
**ACRG#5**	mesenchymal	MSS/EMT	0.19	negative	MSS	preserved	diffuse	poorly differentiated adenocarcinoma	III
**ACRG#6**	non-mesenchymal	MSI	0.24	negative	MSS	loss	diffuse	signet ring cell carcinoma	III
**ACRG#7**	mesenchymal	MSS/EMT	0.3	negative	MSS	preserved	diffuse	poorly differentiated adenocarcinoma	IV
**ACRG#8**	mesenchymal	MSS/EMT	0.12	negative	MSS	preserved	diffuse	poorly differentiated adenocarcinoma	IV
**ACRG#9**	mesenchymal	MSS/EMT	0.2	negative	MSS	preserved	intestinal	moderately differentiated adenocarcinoma	III
**ACRG#10**	non-mesenchymal	MSS/p53 active	0.03	negative	MSS	preserved	intestinal	moderately differentiated adenocarcinoma	IB
**ACRG#11**	non-mesenchymal	MSI	-0.7	negative	MSI-high	loss	diffuse	moderately differentiated adenocarcinoma	IB
**ACRG#12**	non-mesenchymal	MSS/p53 active	-0.3	negative	MSS	preserved	intestinal	moderately differentiated adenocarcinoma	IB
**ACRG#13**	non-mesenchymal	MSS/p53 inactive	-0.44	negative	MSS	preserved	diffuse	poorly differentiated adenocarcinoma	III
**ACRG#14**	non-mesenchymal	MSI	-0.27	negative	MSI-high	loss	diffuse	moderately differentiated adenocarcinoma	II
**ACRG#15**	non-mesenchymal	MSS/p53 active	-0.28	negative	MSS	preserved	diffuse	poorly differentiated adenocarcinoma	III
**ACRG#16**	non-mesenchymal	MSS/p53 active	-0.15	negative	MSS	preserved	diffuse	poorly differentiated adenocarcinoma	III
**ACRG#17**	non-mesenchymal	MSS/p53 inactive	-0.15	ND	ND	preserved	intestinal	poorly differentiated adenocarcinoma	IV
**ACRG#18**	non-mesenchymal	MSI	-0.52	negative	MSS	loss	intestinal	moderately differentiated adenocarcinoma	IB
**ACRG#19**	non-mesenchymal	MSS/p53 inactive	-0.08	negative	MSS	preserved	intestinal	moderately differentiated adenocarcinoma	III
**ACRG#20**	non-mesenchymal	MSS/p53 inactive	-0.49	negative	MSS	preserved	intestinal	moderately differentiated adenocarcinoma	III
**ACRG#21**	non-mesenchymal	MSI	-0.72	negative	MSS	loss	mixed	moderately differentiated adenocarcinoma	III
**ACRG#22**	non-mesenchymal	MSS/p53 active	0.01	negative	MSS	preserved	intestinal	well differentiated adenocarcinoma	II
**ACRG#23**	non-mesenchymal	MSS/p53 inactive	-0.24	positive	MSS	preserved	diffuse	poorly differentiated adenocarcinoma	IV
**ACRG#24**	non-mesenchymal	MSS/p53 inactive	0.01	negative	MSS	preserved	diffuse	poorly differentiated adenocarcinoma	III
**ACRG#25**	non-mesenchymal	MSI	0.02	negative	MSS	preserved	diffuse	poorly differentiated adenocarcinoma	IV
**ACRG#26**	mesenchymal	MSS/EMT	0.26	negative	MSS	preserved	diffuse	poorly differentiated adenocarcinoma	III
**ACRG#27**	non-mesenchymal	MSS/p53 inactive	-0.58	negative	MSI-high	preserved	diffuse	poorly differentiated adenocarcinoma	II
**ACRG#28**	non-mesenchymal	MSS/p53 active	0.06	negative	MSS	preserved	diffuse	poorly differentiated adenocarcinoma	IV
**ACRG#29**	non-mesenchymal	MSS/p53 inactive	-0.57	negative	MSS	preserved	intestinal	moderately differentiated adenocarcinoma	III
**ACRG#30**	non-mesenchymal	MSI	0.49	negative	MSS	preserved	mixed	moderately differentiated adenocarcinoma	III
**ACRG#31**	non-mesenchymal	MSI	-0.58	ND	MSI-high	preserved	intestinal	poorly differentiated adenocarcinoma	IB
**ACRG#32**	non-mesenchymal	MSI	-0.55	negative	MSS	preserved	intestinal	well differentiated adenocarcinoma	IB
**ACRG#33**	non-mesenchymal	MSS/p53 active	-0.09	negative	MSS	preserved	intestinal	moderately differentiated adenocarcinoma	III
**ACRG#34**	non-mesenchymal	MSS/p53 active	-0.2	negative	MSS	preserved	diffuse	poorly differentiated adenocarcinoma	III
**ACRG#35**	non-mesenchymal	MSS/p53 active	-0.07	negative	MSS	preserved	intestinal	poorly differentiated adenocarcinoma	IV
**ACRG#36**	non-mesenchymal	MSS/p53 inactive	-0.01	negative	MSS	preserved	diffuse	signet ring cell carcinoma	IV
**ACRG#37**	non-mesenchymal	MSS/p53 active	-0.92	negative	MSI-high	preserved	intestinal	moderately differentiated adenocarcinoma	IV
**ACRG#38**	non-mesenchymal	MSI	-0.29	negative	MSS	loss	diffuse	poorly differentiated adenocarcinoma	III
**ACRG#39**	mesenchymal	MSS/EMT	-0.01	negative	MSS	preserved	diffuse	signet ring cell carcinoma	III
**ACRG#40**	non-mesenchymal	MSI	-0.6	negative	MSI-high	loss	intestinal	moderately differentiated adenocarcinoma	IB
**ACRG#41**	non-mesenchymal	MSS/p53 active	-0.46	negative	MSS	preserved	intestinal	poorly differentiated adenocarcinoma	III
**ACRG#42**	**non-mesenchymal**	**MSS/EMT**	**0.12**	**negative**		**loss**	**diffuse**	**signet ring cell carcinoma**	**IV**
**ACRG#43**	non-mesenchymal	MSS/p53 active	-0.22	negative	MSS	preserved	intestinal	moderately differentiated adenocarcinoma	IV
**ACRG#44**	non-mesenchymal	MSI	-0.28	negative	MSS	loss	intestinal	moderately differentiated adenocarcinoma	IV
**ACRG#45**	non-mesenchymal	MSS/p53 active	0.12	positive	MSS	preserved	intestinal	poorly differentiated adenocarcinoma	IV
**ACRG#46**	mesenchymal	MSS/EMT	0.32	negative	MSS	preserved	diffuse	poorly differentiated adenocarcinoma	IV
**ACRG#47**	non-mesenchymal	MSS/p53 inactive	-0.58	negative	MSS	preserved	intestinal	moderately differentiated adenocarcinoma	II
**ACRG#48**	mesenchymal	MSS/EMT	0.34	negative	MSS	preserved	diffuse	signet ring cell carcinoma	III
**ACRG#49**	**non-mesenchymal**	**MSS/EMT**	**-0.44**	**negative**		**preserved**	**diffuse**	**signet ring cell carcinoma**	**IV**
**ACRG#50**	non-mesenchymal	MSS/p53 active	-0.22	positive		preserved	diffuse	poorly differentiated adenocarcinoma	IV
**ACRG#51**	non-mesenchymal	MSI	-0.22	negative	NR24 only MSI	preserved	diffuse	poorly differentiated adenocarcinoma	IV
**ACRG#52**	non-mesenchymal	MSI	-0.43	negative	NR24 only MSI	preserved	intestinal	moderately differentiated adenocarcinoma	III
**ACRG#53**	non-mesenchymal	MSS/p53 active	0.22	negative		preserved	diffuse	mucinous adenocarcinoma	III
**ACRG#55**	non-mesenchymal	MSS/p53 active	-0.34	negative		preserved	diffuse	others	II
**ACRG#56**	non-mesenchymal	MSI	-0.15	negative		preserved	diffuse	mucinous adenocarcinoma	III
**ACRG#57**	non-mesenchymal	MSS/p53 active	0.09	positive		preserved	diffuse	poorly differentiated adenocarcinoma	III
**ACRG#58**	non-mesenchymal	MSI	-0.67	negative		loss	intestinal	moderately differentiated adenocarcinoma	IV
**ACRG#59**	non-mesenchymal	MSI	-0.83	negative		loss	intestinal	poorly differentiated adenocarcinoma	III
**ACRG#60**	non-mesenchymal	MSS/p53 active	0.27	negative		preserved	intestinal	poorly differentiated adenocarcinoma	III
**ACRG#61**	non-mesenchymal	MSI	-0.27	294	MSI-high	loss	00283129	67	
**ACRG#62**	non-mesenchymal	MSS/p53 active	0.22	ND		preserved	diffuse	signet ring cell carcinoma	III
**ACRG#63**	non-mesenchymal	MSS/p53 active	0.01	negative		preserved	intestinal	poorly differentiated adenocarcinoma	III
**ACRG#64**	non-mesenchymal	MSS/p53 active	0.08	negative		preserved	diffuse	poorly differentiated adenocarcinoma	III
**ACRG#65**	mesenchymal	MSS/EMT	-0.07	negative		preserved	intestinal	poorly differentiated adenocarcinoma	III
**ACRG#66**	mesenchymal	MSS/EMT	0.45	negative		preserved	diffuse	signet ring cell carcinoma	IV
**ACRG#67**	non-mesenchymal	MSS/p53 inactive	-0.2	negative		preserved	intestinal	moderately differentiated adenocarcinoma	III
**ACRG#68**	mesenchymal	MSS/EMT	0.29	negative		loss	diffuse	signet ring cell carcinoma	IV
**ACRG#69**	non-mesenchymal	MSI	-0.7	negative		loss	mixed	poorly differentiated adenocarcinoma	III
**ACRG#70**	non-mesenchymal	MSS/p53 active	-0.44	negative		preserved	intestinal	papillary adenocarcinoma	II
**ACRG#71**	non-mesenchymal	MSS/p53 active	0.24	positive		preserved	diffuse	poorly differentiated adenocarcinoma	III

### Validation of mesenchymal subtype in the ARTIST cohort

**Figure 3:**
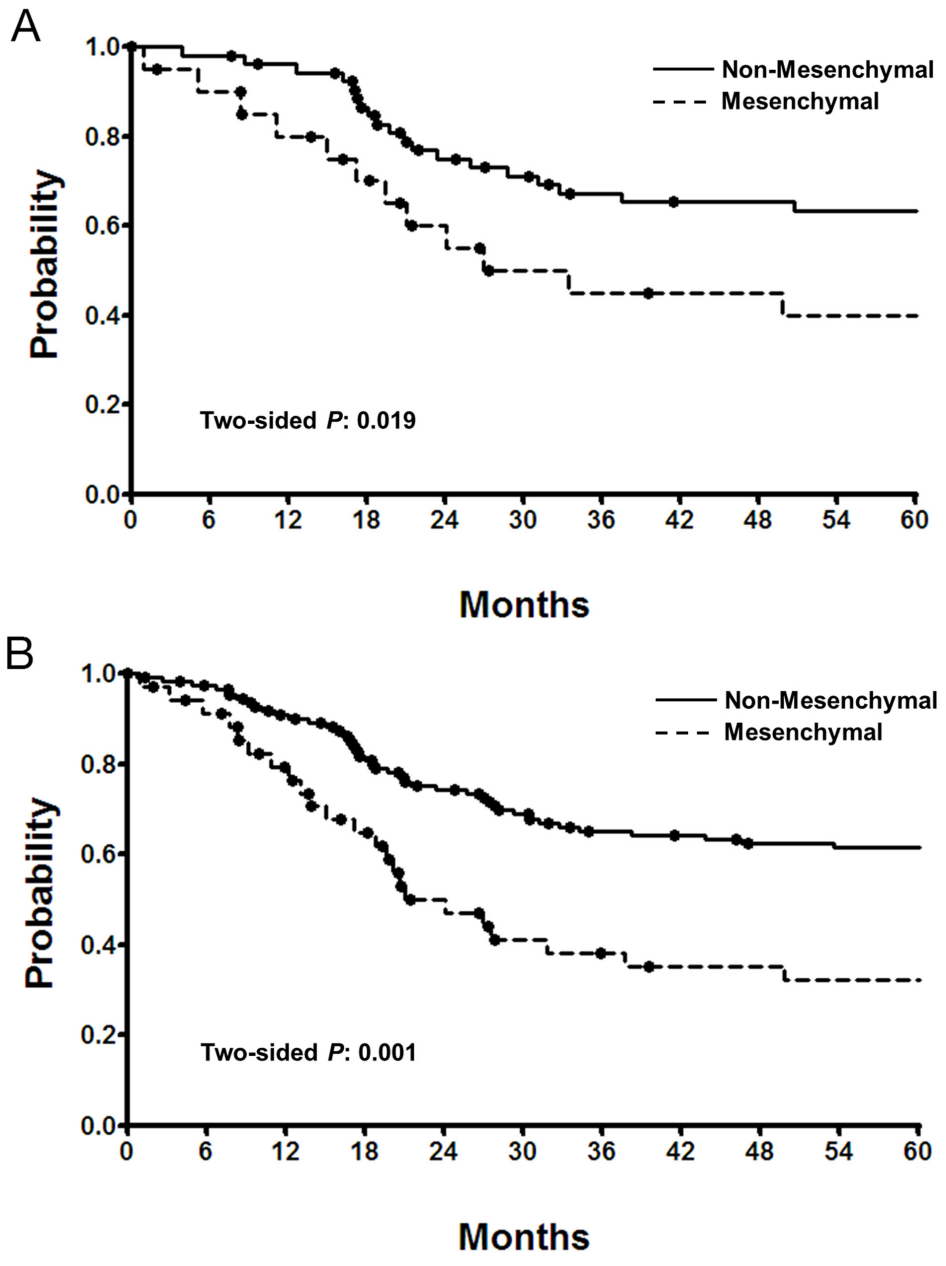
**(A)** Overall survival of the ARTIST patients (n=73) according to the NanoString mesenchymal scores. Solid line, non-mesenchymal (bottom 75% scores). **(B)** Overall survival of all (ARTIST/ACRG combined) patients (n=143) according to the NanoString mesenchymal scores. Solid line, non-mesenchymal (bottom 75% scores); dotted line, mesenchymal (top 25% scores).

In order to validate the mesenchymal subtype, we tested the gene set in 73 samples from the ARTIST cohort. Using the top quartile of the 71-gene mesenchymal signature, 20 of 73 patients predicted to have mesenchymal subtype tumors. The proportion of the mesenchymal subtype, which was equivalent to MSS/EMT, was within our previously reported range. As shown in Figure 3A, patients with the mesenchymal subtype had significantly worse survival compared to non-mesenchymal subtype in the ARTIST cohort (P=0.019).

When combining the two datasets, the comparison of clinical characteristics between mesenchymal and non-mesenchymal subtypes revealed that GC patients with mesenchymal tumors were more likely to have diffuse type disease, GC involving the whole stomach, poorly-differentiated or signet ring cell carcinoma, and MSI low disease (Table 3). Overall survival was significantly shorter in the mesenchymal subtype (hazard ratio [HR], 2.262; 95% confidence interval [CI], 1.410 to 3.636; P=0.001; Figure 3B). In regression analysis with clinical characteristics as covariates, only the mesenchymal subtype (HR, 2.045; 95% CI, 1.205 to 3.472; P=0.008) was independently related to shorter survival. To investigate whether interactions between these clinical characteristics were related to this probability, a stepwise Cox model was used. Again, only the mesenchymal subtype was significantly associated with survival.

**Table 3:** Mesenchymal versus non-mesenchymal subtypes in gastric cancer

	Non-mesenchymal (n=110)	Mesenchymal (n=33)	P
Median age, years	60 (range, 25-78)	56 (range, 36-75)	0.061
Male gender	79 (72%)	19 (58%)	0.095
Tumor location: cardia/body/antrum *vs*. whole			0.041
Cardia	2 (2%)	0	
Body	41 (37%)	14 (42%)	
Antrum	46 (42%)	7 (21%)	
Whole stomach	21 (19%)	12 (36%)	
Tumor grade: PD/signet ring cell *vs*. others			0.012
Well or moderate	22 (20%)	1 (3%)	
Poorly differentiated tubular	44 (40%)	11 (33%)	
Signet ring cell	29 (26%)	21 (64%)	
Mucinous	11 (10%)	0	
Others or unavailable	4 (4%)	0	
Lauren classification			0.001
Intestinal	41 (37%)	1 (3%)	
Diffuse	59 (54%)	30 (91%)	
Mixed or indeterminate	10 (9%)	2 (6%)	
Tumor stage			0.460
I or II	39 (35%)	12 (36%)	
III or IV	71 (65%)	21 (64%)	
EBV positivity	1 (1%)	2 (6%)	0.001
MSI high	24 (22%)	2 (6%)	0.035
Lymphovascular invasion	74 (67%)	20 (61%)	0.961
Perineural invasion	41 (37%)	12 (36%)	0.767

## DISCUSSION

Because of the distinct clinicopathologic features of the MSS/EMT subtype in GC, it is considered clinically meaningful to stratify GC subtypes based on genomic or transcriptional aberrations. According to our previous study [8], patients with the MSS/EMT subtype have a more aggressive natural history including high recurrence rate, predilection for peritoneal seeding at the first site of recurrence, younger age at diagnosis, and extremely poor survival. Hence, we hypothesized that treatment strategies and/or clinical trial designs for this particular subset of GC patients should be treated differently. Likewise, for a successful GC clinical trial involving specific molecularly targeted agents, it may be crucial to account for the mesenchymal subtype to enhance treatment outcome. In addition, in this era of immunotargeted therapy, stratification according to EMT may be increasingly important in terms of tumor immune infiltrates or responsiveness to immune checkpoint inhibitors [13].

The use of accurate molecular biomarkers to stratify patients with GC may lead not only to personalized treatment, but also to potential reductions in healthcare costs. Recently, a growing body of evidence supports 4 main molecular subtypes of GC distinguished by gene expression profiling [6-10]. Although the use of tumor biomarkers has been proposed for decades, the discovery of specific genetic or protein biomarkers has been fundamentally complex because of the technical nature of comprehensive expression platforms, limitations in multiplex clinical assay development and, most importantly, an incomplete understanding of tumor biology. Most clinical specimens are FFPE tissues, particularly in cancer patients, and extensive RNA sequencing may not be feasible in clinically available specimens. We previously demonstrated that targeted profiling by the NanoString nCounter assay is a feasible and reliable method that can be readily used with FFPE specimens [14-16]. Importantly, in the present study, we successfully constructed a gene signature derived from conventional gene expression profiling and cross-validated in an independent GC cohort. The concordance rate between NanoString and conventional gene expression profiling for identifying the MSS/EMT subtype was extremely high: only 2 discordant cases were found among 70 specimens. The identified mesenchymal subtype showed aggressive tumor behaviors such as diffuse type disease, GC involving the whole stomach, poorly-differentiated or signet ring cell carcinoma, MSI low, and significantly shorter survival. The distinct molecular and clinical features indicate that the mesenchymal subtype arises from different transformed stem or progenitor cells, with distinct biologic properties.

Previous studies suggested that substantial improvement in the treatment of GC can be achieved by using individualized therapy strategies [17], including the identification of genetic alterations and the study of molecular biology of therapeutic agents. Recently, antibodies directed against immune checkpoint proteins have shown therapeutic efficacy in a number of cancer types [18]. In limited feasibility studies [19], immunotargeted therapy also showed promising antitumor activity in GC. The efficacy of these immune checkpoint blockades vary among different tumor types, and an increased understanding of these differences may enhance the efficacy of this treatment modality. Attention is now focused on the identification of predictive biomarkers to select patients for immunotargeted therapy, although currently no single immunologic or tumoral characteristic in a patient has been found to solely determine response to an immunotherapeutic agent. One of the potential biomarkers is an inflamed tumor phenotype [20], as a non-inflamed tumor microenvironment may predict the resistance to immunotargeted therapy. EMT, or mesenchymal subtype, is highly associated with the inflammatory tumor microenvironment, independent of tumor mutation burden [13].

Interestingly, two MSS/EMT tumors had non-mesenchymal NanoString genotypes, likely because of intratumoral heterogeneity. Given the molecular tumor status is generally detected in a small fraction of the primary tumor, heterogeneity may limit treatment decisions based on a single biomarker test [21]. From a practical perspective, careful selection of the most poorly-differentiated area for RNA extraction would make it unlikely that this intratumoral heterogeneity, when present, will lead to incorrect results. Another limitation of the present study is the potential ethnic differences in GC patients. It is well known that significant geographic variation in the GC incidence exists, with the highest rates being reported in East Asian countries including Korea, and survival outcomes also differ considerably between Western and Asian countries. This discrepancy may be related to different diagnostic or treatment policies, and different tumor biology [22]. The different patterns of GC between Western and Asian countries are quite apparent, and thus our results warrant validation in different ethnic groups. However, our main focus has been the identification of a distinct, mesenchymal GC subtype with very poor prognosis, and it is clear that the detection of molecular subtypes may enable the stratification of patients with high risk and development of the most appropriate treatment. Potential biological differences between the subtypes may suggest different therapeutic approaches with different molecular targets.

## MATERIALS AND METHODS

The ACRG cohort consisted of 300 primary GC specimens that were procured at the time of curative or palliative gastrectomy at Samsung Medical Center (SMC, Seoul, Korea) between 2004 and 2007, and frozen at -80°C as previously reported [8]. The study protocol was reviewed and approved by the SMC Institutional Review Board (IRB No. 2010-12-088). All participating subjects provided written informed consent after being informed about the purpose and investigational nature of the study. Cases were selected based on the following criteria: histologically confirmed adenocarcinoma arising from the stomach; surgical resection of primary GC; aged 18 years or older; complete pathological, surgical, treatment and survival follow-up data. Primary GC tissues were used for genomic analysis. Of the 300 patients, 70 tumor specimens were randomly selected based on the availability of tissue specimens. For validation, we selected 73 patients from the ARTIST [11], a phase III trial comparing adjuvant chemotherapy with chemoradiotherapy in 458 GC patients, in whom tissue specimens were available and sufficient for RNA extraction. In both cohorts, all tumor specimens were prepared from primary surgical specimen. Clinical characteristics of the patients are listed in Table 1. All patients were of Korean ethnicity.

### RNA preparation

Hematoxylin and Eosin stain was performed on one tumor section per patient and tumors were reviewed by a pathologist (KMK) for tumor purity. Samples containing <50% tumor was discarded from the study. The tumor component was macro-dissected from 2 x 5μm formalin-fixed paraffin-embedded (FFPE) tissue sections or fresh frozen samples, and RNA was extracted using the RNeasy FFPE Extraction kit or QIAamp DNA Mini Kit (Qiagen, Hilden, Germany) according to the manufacturer’s instructions. Sample RNA was quantified using Qubit 2.0 Flourometer with the Broad Range RNA kit using the standard protocol. Samples containing <20 ng/μl total RNA were not tested in the NanoString assay. Where available, more tissue for these samples were ordered, re-extracted, and those containing 20 ng/ul or greater were tested in the NanoString assay.

### Gene expression profiling: Affymetrix microarray

For training the algorithm for gene selection for the signature, we used the previously published dataset (accessed via https://www.ncbi.nlm.nih.gov/geo/query/acc.cgi?acc=GSE62254); RNA was extracted from tumors according to the manufacturer’s protocol (Affymetrix, Santa Clara, CA, USA) [8]. We used Affymetrix Human Genome U133plus 2.0 Array for gene expression profiling and processed the raw files using standard Affymetrix software including RMA normalization.system.

### Gene expression profiling: NanoString

In the NanoString assay, we included 584 genes that were previously published to define the 4 subtypes, including 15 housekeeping and 14 technical control genes. The NanoString assays were performed following the standard protocol ‘Setting up 12 nCounter Assays (MAN-C0003-03, 2008-2013)’. Hybridization incubations were performed between 17 and 18 h. Cartridges were either read immediately or stored dark (in aluminum foil) at 4°C until reading. All cartridges were read within 2 days of preparation on the AZ GEN2 Digital Analyzer station with high resolution selected. Data were processed using nCounter PanCancer pathways (Supplementary Table 1), and were normalized by dividing the raw counts by the geometric mean of the manufacturer-defined housekeeping genes and transforming into a log10 scale.

### Gene expression cross-platform concordance filter

For each gene, we calculated the correlation between the gene expression level on the NanoString platform and on the microarray platform in the training set (n=70). Following inspection of the distribution of correlations (Supplementary Figure 1) we chose a cutoff of 0.4 correlation to select genes that were concordant between the two platforms. The genes remaining in the signature are represented in Supplementary Table 2. Original up (UP) and down (DN) arms of the EMT signature were previously defined [23]. UP/DN refers to up/down regulation of genes at a pre-defined significance levels in a panel of solid cell lines defined as Epithelial or Mesenchymal using levels of CDH1 and VIM.

### Gene signature analysis

We calculated the mesenchymal signature on the NanoString platform using the average of the genes in our previously defined GC mesenchymal signature [8], down-selected to genes present on the NanoString platform, and with cross-platform concordance as defined in the previous section.

### Statistical analysis

The primary endpoint of the present study was the identification and validation of a mesenchymal gene signature in GC. The secondary endpoint was survival, defined as the time between the date of surgery and the date of death. Survival data were updated at the time of analyses (May 2016), and analyzed using a Cox regression model. Baseline characteristics were compared using chi-square or Fisher’s exact test. We used Spearman correlation for pairwise correlations between continuous variables. The significance levels were set at alpha=0.05. All analyses were performed using either the Matlab package including the Statistics toolbox (Mathworks, Natick, MA, USA) or R for Windows, v2.15 (R Core Team, Vienna, Austria; http://www.Rproject.org).

## CONCLUSION

In the present study, we evaluated the gene signature of GC for mesenchymal subtype using a targeted NanoString gene expression, and validated the findings in an independent GC patient cohort. We found a 71-gene signature for mesenchymal GC with a high concordance rate. Because GC is considered a heterogeneous disease, it appears unlikely that one genomic and/or transcriptomal change will be uniformly defined. Therefore, a panel of biomarkers (i.e., gene signature) may enable more accurate prediction than a single biomarker. The results of the present study support the use of gene expression profiling analyses for the stratification of GC patients. Our results also provide further insight into the molecular heterogeneity of GC, and set the foundation for more detailed investigations, leading to the identification of a patient subset for novel, individualized therapy.

## SUPPLEMENTARY MATERIALS FIGURE AND TABLES





## References

[1] Torre LA, Bray F, Siegel RL, Ferlay J, Lortet-Tieulent J, Jemal A (2015). Global cancer statistics, 2012. CA Cancer J Clin.

[2] Bang YJ, Van Cutsem E, Feyereislova A, Chung HC, Shen L, Sawaki A, Lordick F, Ohtsu A, Omuro Y, Satoh T, Aprile G, Kulikov E, Hill J (2010). Trastuzumab in combination with chemotherapy versus chemotherapy alone for treatment of HER2-positive advanced gastric or gastro-oesophageal junction cancer (ToGA): a phase 3, open-label, randomised controlled trial. Lancet.

[3] Fuchs CS, Tomasek J, Yong CJ, Dumitru F, Passalacqua R, Goswami C, Safran H, dos Santos LV, Aprile G, Ferry DR, Melichar B, Tehfe M, Topuzov E (2014). Ramucirumab monotherapy for previously treated advanced gastric or gastro-oesophageal junction adenocarcinoma (REGARD): an international, randomised, multicentre, placebo-controlled, phase 3 trial. Lancet.

[4] Wilke H, Muro K, Van Cutsem E, Oh SC, Bodoky G, Shimada Y, Hironaka S, Sugimoto N, Lipatov O, Kim TY, Cunningham D, Rougier P, Komatsu Y (2014). Ramucirumab plus paclitaxel versus placebo plus paclitaxel in patients with previously treated advanced gastric or gastro-oesophageal junction adenocarcinoma (RAINBOW): a double-blind, randomised phase 3 trial. Lancet Oncol.

[5] Kim SM, Park SH (2015). Chemotherapy beyond second-line in advanced gastric cancer. World J Gastroenterol.

[6] Wong SS, Kim KM, Ting JC, Yu K, Fu J, Liu S, Cristescu R, Nebozhyn M, Gong L, Yue YG, Wang J, Ronghua C, Loboda A (2014). Genomic landscape and genetic heterogeneity in gastric adenocarcinoma revealed by whole-genome sequencing. Nat Commun.

[7] Cancer Genome Atlas Research Network (2014). Comprehensive molecular characterization of gastric adenocarcinoma. Nature.

[8] Cristescu R, Lee J, Nebozhyn M, Kim KM, Ting JC, Wong SS, Liu J, Yue YG, Wang J, Yu K, Ye XS, Do IG, Liu S (2015). Molecular analysis of gastric cancer identifies subtypes associated with distinct clinical outcomes. Nat Med.

[9] Chen K, Yang D, Li X, Sun B, Song F, Cao W, Brat DJ, Gao Z, Li H, Liang H, Zhao Y, Zheng H, Li M (2015). Mutational landscape of gastric adenocarcinoma in Chinese: implications for prognosis and therapy. Proc Natl Acad Sci U S A.

[10] Kakiuchi M, Nishizawa T, Ueda H, Gotoh K, Tanaka A, Hayashi A, Yamamoto S, Tatsuno K, Katoh H, Watanabe Y, Ichimura T, Ushiku T, Funahashi S (2014). Recurrent gain-of-function mutations of RHOA in diffuse-type gastric carcinoma. Nat Genet.

[11] Lee J, Lim do H, Kim S, Park SH, Park JO, Park YS, Lim HY, Choi MG, Sohn TS, Noh JH, Bae JM, Ahn YC, Sohn I (2012). Phase III trial comparing capecitabine plus cisplatin versus capecitabine plus cisplatin with concurrent capecitabine radiotherapy in completely resected gastric cancer with D2 lymph node dissection: the ARTIST trial. J Clin Oncol.

[12] Park SH, Sohn TS, Lee J, Lim do H, Hong ME, Kim KM, Sohn I, Jung SH, Choi MG, Lee JH, Bae JM, Kim S, Kim ST (2015). Phase III trial to compare adjuvant chemotherapy with capecitabine and cisplatin versus concurrent chemoradiotherapy in gastric cancer: final report of the adjuvant chemoradiotherapy in stomach tumors trial, including survival and subset analyses. J Clin Oncol.

[13] Lou Y, Diao L, Cuentas ER, Denning WL, Chen L, Fan YH, Byers LA, Wang J, Papadimitrakopoulou VA, Behrens C, Rodriguez JC, Hwu P, Wistuba II (2016). Epithelial-mesenchymal transition is associated with a distinct tumor microenvironment including elevation of inflammatory signals and multiple immune checkpoints in lung adenocarcinoma. Clin Cancer Res.

[14] Lee J, Sohn I, Do IG, Kim KM, Park SH, Park JO, Park YS, Lim HY, Sohn TS, Bae JM, Choi MG, Lim DH, Min BH (2014). Nanostring-based multigene assay to predict recurrence for gastric cancer patients after surgery. PLoS One.

[15] Kim ST, Lee J, Hong M, Park K, Park JO, Ahn T, Park SH, Park YS, Lim HY, Sun JM, Ahn JS, Ahn MJ, Kim HC (2015). The NEXT-1 (Next generation pErsonalized tX with mulTi-omics and preclinical model) trial: prospective molecular screening trial of metastatic solid cancer patients, a feasibility analysis. Oncotarget.

[16] Kim ST, Do IG, Lee J, Sohn I, Kim KM, Kang WK (2015). The NanoString-based multigene assay as a novel platform to screen EGFR, HER2, and MET in patients with advanced gastric cancer. Clin Transl Oncol.

[17] Lee J, Ou SH (2013). Towards the goal of personalized medicine in gastric cancer--time to move beyond HER2 inhibition. Part II: targeting gene mutations and gene amplifications and the angiogenesis pathway. Discov Med.

[18] Postow MA, Callahan MK, Wolchok JD (2015). Immune checkpoint blockade in cancer therapy. J Clin Oncol.

[19] Muro K, Chung HC, Shankaran V, Geva R, Catenacci D, Gupta S, Eder JP, Golan T, Le DT, Burtness B, McRee AJ, Lin CC, Pathiraja K (2016). Pembrolizumab for patients with PD-L1-positive advanced gastric cancer (KEYNOTE-012): a multicentre, open-label, phase 1b trial. Lancet Oncol.

[20] Gajewski TF, Fuertes M, Spaapen R, Zheng Y, Kline J (2011). Molecular profiling to identify relevant immune resistance mechanisms in the tumor microenvironment. Curr Opin Immunol.

[21] Kim KM, Bilous M, Chu KM, Kim BS, Kim WH, Park YS, Ryu MH, Sheng W, Wang J, Chao Y, Ying J, Zhang S (2014). Human epidermal growth factor receptor 2 testing in gastric cancer: recommendations of an Asia-Pacific task force. Asia Pac J Clin Oncol.

[22] Macdonald JS (2011). Gastric cancer: Nagoya is not New York. J Clin Oncol.

[23] Loboda A, Nebozhyn MV, Watters JW, Buser CA, Shaw PM, Huang PS, Van’t Veer L, Tollenaar RA, Jackson DB, Agrawal D, Dai H, Yeatman TJ (2011). EMT is the dominant program in human colon cancer. BMC Med Genomics.

