# Supplementary material for: Development of mesenchymal subtype gene signature for clinical application in gastric cancer
Source: Oncotarget. 2017 Aug 7;8(39):66305–15. doi: 10.18632/oncotarget.19985 (PMC5630413; doi:10.18632/oncotarget.19985)
Supplement: Supplementary file 1 [file oncotarget-08-66305-s001.pdf]

Development of mesenchymal subtype gene signature for clinical application in gastric cancer

SUPPLEMENTARY MATERIALS

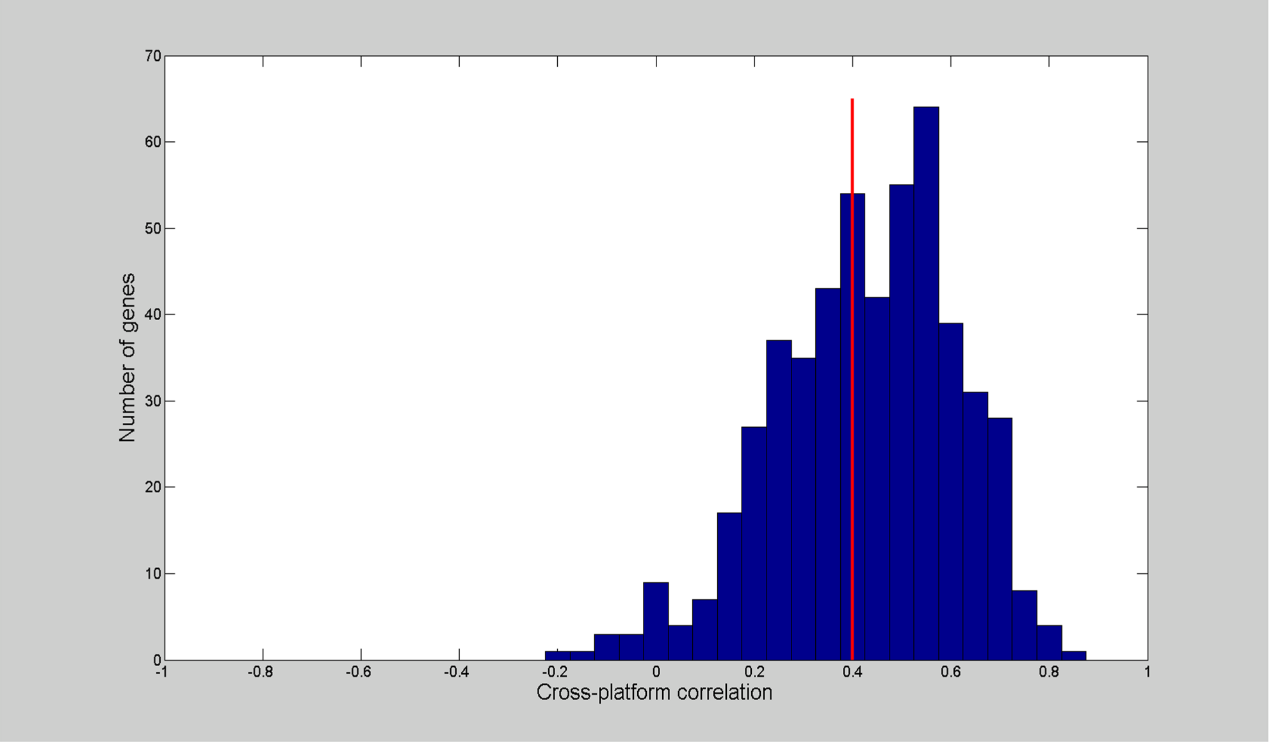

Supplementary Figure 1: Distribution of correlations between the gene expression level on the NanoString and the microarray platform.

Supplementary Table 1: NanoString nCounter PanCancer pathways

See Supplementary File 1

Supplementary Table 2: Genes remained in the signature with a cutoff value of 0.4 correlations between the NanoString and the microarray platforms

EMT up (in alphabetical order)	ADAM23, ADAMTS1, AKAP12, ALPK2, AP1S2, ARMCX1, AXL, BICC1, BNC2, C16orf45, C1S, CLDN11, CMTM3, CTGF, CYBRD1, DIO2, DKK3, DOCK10, DPYSL3, EML1, EVI2A, FAM101B, FAT4, FGF2, FGFR1, FHL1, FLRT2, FSTL1, GLIPR1, GNB4, GNG11, HEG1, IGFBP7, JAM3, LEPREL1, LHFP, MAP1B, MMP2, MRAS, MSRB3, NDN, NEGR1, NEXN, NID1, PLAGL1, PMP22, PRKD1, PTGIS, RBMS3, RECK, RFTN1, SRPX, TCF4, TMEM47, TTC28, UCHL1, VIM, ZEB1, ZEB2, ZFPM2
EMT down	C19orf21, CLDN4, CLDN7, DSC2, GOLT1A, LIPG, MAP7, PAK6, PKP3, SH2D3A, SPINT1
